# Primary percutaneous coronary intervention in nonagenarians: is it worthwhile?

**DOI:** 10.1186/s12872-020-01833-2

**Published:** 2021-01-13

**Authors:** Mohammed M. N. Meah, Tobin Joseph, Wern Yew Ding, Matthew Shaw, Jonathan Hasleton, Nick D. Palmer, Periaswamy Velavan, Suneil K. Aggarwal

**Affiliations:** Liverpool Heart and Chest Hospital, Thomas Drive, Liverpool, L14 3PE UK

**Keywords:** Myocardial infarction, Nonagenarian, Primary PCI

## Abstract

**Background:**

Previous studies have demonstrated the feasibility of primary percutaneous coronary intervention (PPCI) in carefully selected nonagenarians. Although current guidelines recommend immediate revascularization in patients with ST elevation myocardial infarction (STEMI) it remains unclear whether PPCI reduces mortality in nonagenarians. The objective of this study is to compare mortality in nonagenarians presenting via the PPCI pathway who undergo coronary intervention, versus those who are managed medically.

**Methods and results:**

A total of 111 consecutive nonagenarians who presented to our tertiary center via the PPCI pathway between July 2013 and December 2018 with myocardial infarction were included. Clinical and angiographic details were collected alongside data on all-cause mortality. The final diagnosis was STEMI in 98 (88.3%) and NSTEMI in 13 (11.7%). PPCI was performed in 42 (37.8%), while 69 (62.2%) were medically managed. A significant number of the medically managed cohort had atrial fibrillation (23.2% vs 2.4% *p* = 0.003) and presented with a completed infarct (43.5% vs 4.8% *p* = 0.001). Other baseline and clinical variables were well matched in both groups. There was a trend towards increased 30-day mortality in the medically managed group (40.6% vs 23.8% *p* = 0.07). Kaplan Meier survival analysis demonstrated a significant difference in survival by 3 years (48.1% vs 21.7% *p* = 0.01). This was the case even when those with completed infarcts were excluded (44.3% vs 14.6%, *p* = 0.01).

**Conclusion:**

In this series of selected nonagenarians presenting with acute myocardial infarction, those undergoing PPCI appeared to have a lower mortality compared to those managed medically.

## Background

The Office for National Statistics reported a 28% rise in nonagenarians between 2007 and 2017 in the UK [1]. They are however underrepresented in the available literature on primary percutaneous coronary intervention (PPCI) and indeed actively excluded from most randomized control trials. They can be amongst the frailest of patients and as such warrant careful consideration on whether the risks of coronary intervention outweigh the potential benefits.

A review article looking at the pros and cons of any coronary intervention in nonagenarians found, when carefully selected, the procedure is safe [2]. It suggested, rather than using age as a discriminating factor, a more detailed assessment of frailty is required. With increasing life-expectancy it is not surprising that registry data over the last 10 years indicate Cardiologists are managing more nonagenarians invasively. The percentage of invasively managed patients in this population has risen from 23% of ST elevation myocardial infarction (STEMI) cases to 31% [3, 4].

A multicenter retrospective study conducted in France recruited 145 nonagenarians treated with PPCI, demonstrating survival rates at 1 year of 53% [5]. Retrospective studies comparing longer term outcomes between patients undergoing PPCI compared to medical management are sparse [6]. The largest followed 73 patients for 2 years and demonstrated a trend towards increased survival in the PPCI cohort that did not reach statistical significance [7]. The present study seeks to describe our experience of managing nonagenarians presenting with acute myocardial infarction via the PPCI pathway in a large tertiary centre with a large number of PPCI activations (approximately 1600/year).

## Purpose

The primary objective of this study is to compare long term survival in nonagenarians managed medically against those who underwent PPCI. Secondary analysis will focus on factors that predicted 30-day mortality in those patients managed with coronary intervention.

## Methods

We performed a retrospective single centre study of consecutive nonagenarians who arrived at our tertiary centre as a PPCI activation between July 2013 and December 2018. Patients were identified from a prospectively collected database of over 12,000 activations for PPCI. At our centre there is no age restriction for PPCI activation. The inclusion criteria were, symptoms of myocardial ischaemia lasting > 30 min with new significant ST-segment or T wave changes or echo evidence of new regional wall motion abnormality in patients with left bundle branch block or a paced rhythm. Patients were divided into those who had immediate PCI and those managed medically. Data on demographics, clinical condition on arrival, angiographic details, symptom/door to balloon time, 30-day and longer-term survival were manually collected from electronic records.

The third universal definition was used for myocardial infarction [8]. STEMI required ST-segment elevation > 1 mm in ≥ 2 contiguous ECG leads. Cardiogenic shock was defined as a systolic blood pressure of < 90 mmHg for a minimum of 30 min or the need to use supportive treatments (inotropes/intra-aortic balloon pump). Successful PCI was defined as the achievement of ≥ TIMI grade 2 flow and residual stenosis of < 30% on final angiography. A completed infarct was defined as presentation 12 h after symptom onset with associated pathological Q waves on ECG. Atrio-ventricular block was defined as Mobitz type II or complete heart block.

All patients undergoing PCI were pretreated with dual antiplatelet therapy (aspirin administered by paramedics, P2Y12 inhibitor immediately pre-procedure) and given weight adjusted heparin peri-procedurally to maintain an activated clotting time of > 250 s. Radial access and drug eluting stents were used in all cases. Arrhythmia on admission was documented as new if there was no prior clinical history evident. Data on antiplatelet therapy and anticoagulation on discharge was collected. Every patient had an echo performed during their admission, from which LV function was derived.

### Statistical analysis

Categorical variables are presented as absolute number (percentage); comparisons are made with the chi-squared test or Fisher’s exact test as appropriate. Continuous variables are presented as mean ± standard deviation; comparisons are made using the Wilcoxon Signed Rank test. Follow-up survival is plotted using the Kaplan–Meier method. Additionally, we compared survival to an age and sex matched cohort of nonagenarians taken from the Office for National Statistics (ONS) life tables. Statistical analysis was performed using SAS v9.3 (SAS Institute, Cary, NC). A *p* value of < 0.05 was considered statistically significant.

### Patient and public involvement

The patients and public were not involved in the design, data collection, analysis or preparation of this study.

## Results

There were 157 nonagenarian PPCI activations of which 46 were false activations (29.3%). There were 20 activations in the first 12 months of this study compared to 48 in the last 12 months. Of those presenting with true myocardial infarction (n = 111, 70.7%) the final diagnosis was STEMI in 98 (88.3%) and NSTEMI in 13 (11.7%). PPCI was performed in 42 (37.8%), while 69 (62.2%) were medically managed (MM). Table 1 summarises baseline demographics and clinical status on admission.

**Table 1:** Demographics and co-morbidities

Variables	PPCI patients (n = 42)	MM patients (n = 69)	*P* value
Mean age at operation (years)	92.8 ± 2.5	93.2 ± 2.2	0.22
Female gender, n (%)	21 (50.0)	45 (65.2)	0.11
Assisted living	7 (16.7)	17 (24.6)	0.32
Hypertension	24 (57.1)	45 (65.2)	0.39
Diabetes	5 (11.9)	18 (26.1)	0.07
Chronic kidney disease (e-GFR < 60)	12 (28.6)	25 (36.2)	0.41
History of previous cancer	4 (9.5)	8 (11.6)	> 0.99
History of atrial fibrillation	1 (2.4)	16 (23.2)	**0.003**
Chronic obstructive pulmonary disease	2 (4.8)	1 (1.5)	0.56
Previous stroke	8 (19.1)	15 (21.7)	0.73
Dementia	1 (2.4)	3 (4.4)	> 0.99
Old bundle branch block	6 (14.3)	6 (8.7)	0.37
Pacemaker/Defibrillator	6 (14.3)	3 (4.4)	0.08
Ex-smoker	14 (33.3)	27 (37.1)	0.54
Current smoker	1 (2.4)	5 (7.3)	0.41
*Arrhythmia on admission*			
New atrial fibrillation	9/41 (22.0)	4/53 (7.5)	0.08
New atrio-ventricular block	8 (19.1)	3 (4.4)	**0.019**
*Pre-admission NYHA class*			
1	27 (64.3)	39 (56.5)	0.42
2	12 (28.6)	26 (37.7)	0.33
3	2 (4.8)	4 (5.8)	> 0.99
4	1 (2.4)	0 (0)	0.38

Values in bold demonstrate statistically significant results (*p* < 0.05)

*PPCI* primary percutaneous coronary intervention, *MM* medically managed, *e-GFR *estimated glomerular filtration rate

± values are mean ± standard deviation. Values in brackets are (%)

No difference in age or sex was seen between those managed medically and those undergoing PPCI. There was no statistically significant difference between the number of patients who lived in a residential or nursing home (assisted living 16.7% in PPCI group versus 24.6% in the medically managed group *p* = 0.32). Most patients with a previous history of atrial fibrillation (AF) were managed medically (23.2% vs 2.4% *p* = 0.003), otherwise no other differences were noted in the comorbidities. Interestingly, those who presented with new atrio-ventricular block were more likely to be managed with PCI (19.1% vs 4.4% *p* = 0.019). No difference was seen depending on pre-admission NYHA class. The incidence of previous stroke or dementia in this cohort was low and equally distributed.

Table 2 summarises left ventricular function, admission biochemistry, clinical condition of the patients on admission and 30-day mortality.

**Table 2:** Left ventricular function, biochemistry, clinical condition and outcome

Variables	PPCI patients (n = 42)*	MM patients (n = 69)*	*P* value
*Left ventricular systolic function*	(*n = 38*)	(*n = 61*)	
Normal (EF > 55%),	5 (11.9)	8 (11.6)	> 0.99
Mildly impaired (EF 46–55%)	6 (14.3)	7 (10.1)	0.55
Moderately impaired (EF 36–45%)	16 (38.1)	21 (30.4)	0.41
Severely impaired (EF ≤ 35%)	11 (26.2)	25 (36.2)	0.27
CK-MB on admission (µg/L)	53.0 ± 89.0	66.9 ± 101.3	0.24
Total Cholesterol (mmol/L),	4.3 ± 1.5	4.2 ± 1.1	0.97
Sodium (mmol/L)	135.9 ± 3.6	135.6 ± 4.1	0.32
Potassium (mmol/L)	4.5 ± 0.5	4.6 ± 0.6	> 0.99
Urea (mmol/L)	9.1 ± 3.3	9.8 ± 4.4	0.93
Creatinine (µmol/L)	109.3 ± 37.9	123.5 ± 72.1	0.54
Glucose (mmol/L)	9.2 ± 3.9	8.5 ± 4.5	0.33
Haemoglobin (g/L)	118.7 ± 16.5	117.7 ± 14.3	0.99
Heart rate on admission (beats/min)	74.3 ± 17.3	79.9 ± 19.0	0.14
Systolic blood pressure (mmHg)	119.5 ± 14.1	120.8 ± 19.1	0.15
Cardiogenic shock	4 (9.5)	6 (8.7)	> 0.99
Completed Infarct	2 (4.8)	30 (43.5)	** < 0.001**
NSTEMI	1 (2.4)	12 (17.4)	**0.017**
STEMI	41 (97.6)	57 (82.6)	**0.017**
Anterior	19 (46.3)	26 (45.6)	0.94
Inferior	17 (41.5)	21 (36.8)	0.64
Lateral	4 (9.8)	7 (12.3)	0.76
Posterior	1 (2.4)	3 (5.3)	0.64
Direct admission via ambulance	31 (73.8)	41 (59.4)	0.12
Transfer from DGH	11 (26.2)	28 (40.6)	0.12
Angiogram Performed	42 (100)	9 (13.0)	< 0.001
*30-day mortality*	10 (23.8)	28 (40.6)	0.07
*BCIS PCI 30-day mortality score*	22.9 ± 17.4	24.0 ± 16.6	0.17

Values in bold demonstrate statistically significant results (*p* < 0.05)

*PPCI* primary percutaneous coronary intervention, *MM* medically managed, *EF* ejection fraction, *DGH* District general hospital, *BCIS* British Cardiovascular Intervention Society

± values are mean ± standard deviation. Values in brackets are (%)

^*^Unless otherwise stated

Both groups had comparable blood test results and left ventricular function. A significant proportion of the medically managed group presented with a completed infarct (43.5% vs 4.8% *p* = 0.001). There was no difference in the number of patients who arrived in cardiogenic shock (9.5% vs 8.7% *p* > 0.99) or in acute heart failure. In 13% of the medically managed cases although patients were taken into the lab with the intention to PCI, revascularisation was deemed unfeasible due to complex coronary anatomy. More patients who presented with NSTEMI were managed medically (17.4% vs 2.4% *p* = 0.017). Infarct location determined by ECG did not correlate with the decision to perform PPCI.

In total 65% of patients were admitted directly via the ambulance service versus 35% who presented or were taken to a peripheral hospital first. Of those that underwent PPCI only a quarter came from peripheral hospitals with 73.8% being brought directly via the ambulance service.

There was a trend towards increased 30-day mortality in the medically managed group (40.6% vs 23.8% *p* = 0.07). We calculated the BCIS PCI 30-day mortality risk score in every patient using admission variables and found no significant difference in the mean score between groups (22.9% vs 24.0% *p* = 0.17).

**Fig. 1:**
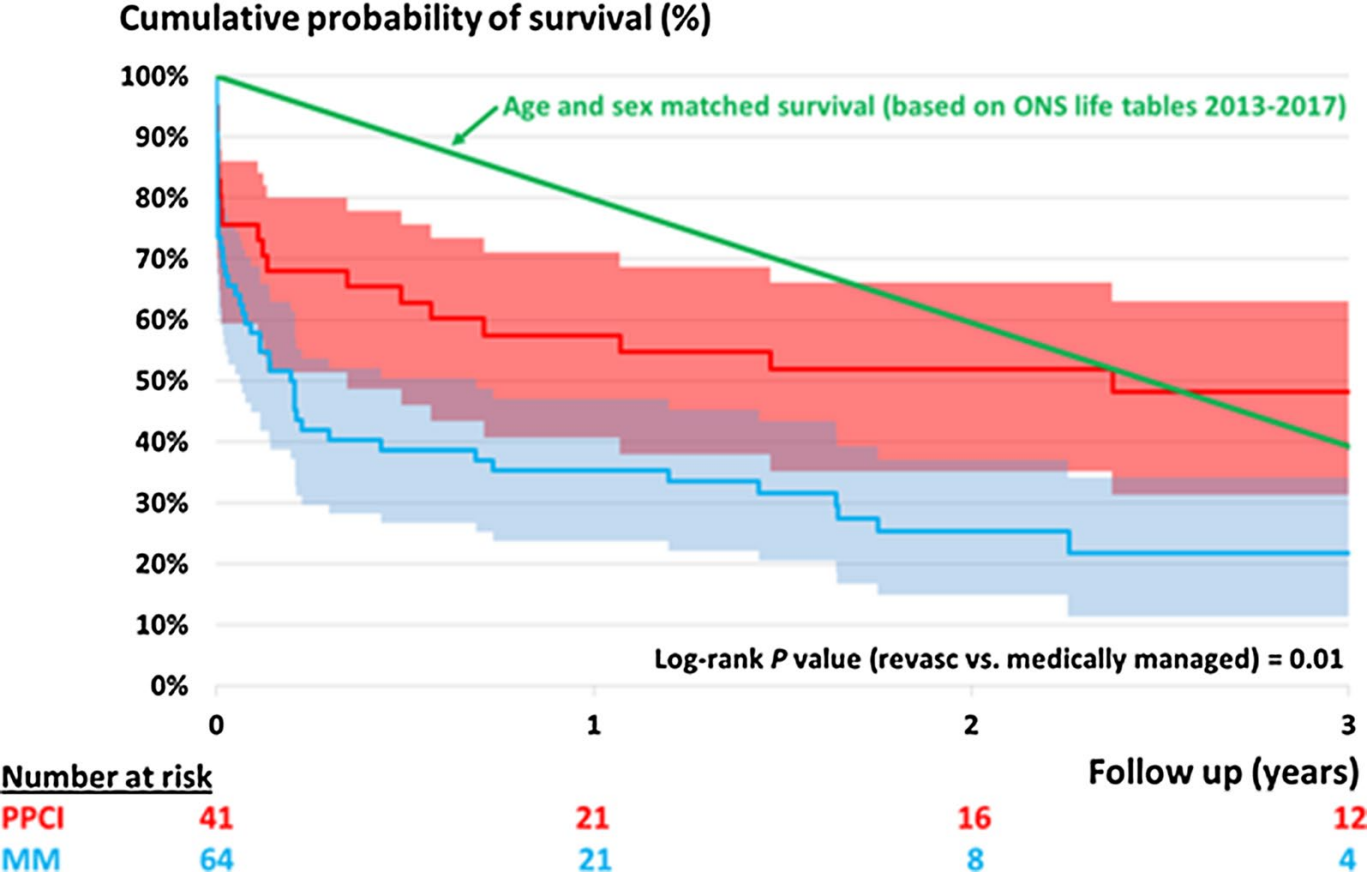
Kaplan Meier for all-cause mortality

Figure 1 shows the Kaplan Meier chart for all-cause mortality.

A statistically significant difference in survival was reached by 3 years (48.1% vs 21.7% *p* = 0.01). Compared to the mortality of an age/sex matched population of nonagenarians (taken from the ONS life tables), survival at 3 years is equivalent in the PPCI cohort and worse in those managed medically at 3 years.

A total of 32 patients presented with completed infarcts, of which 30 were managed medically. We repeated a Kaplan Meier analysis excluding these patients to directly compare outcomes in patients with acute STEMI in relation to treatment strategy (PCI group n = 40, MM group n = 39).

**Fig. 2:**
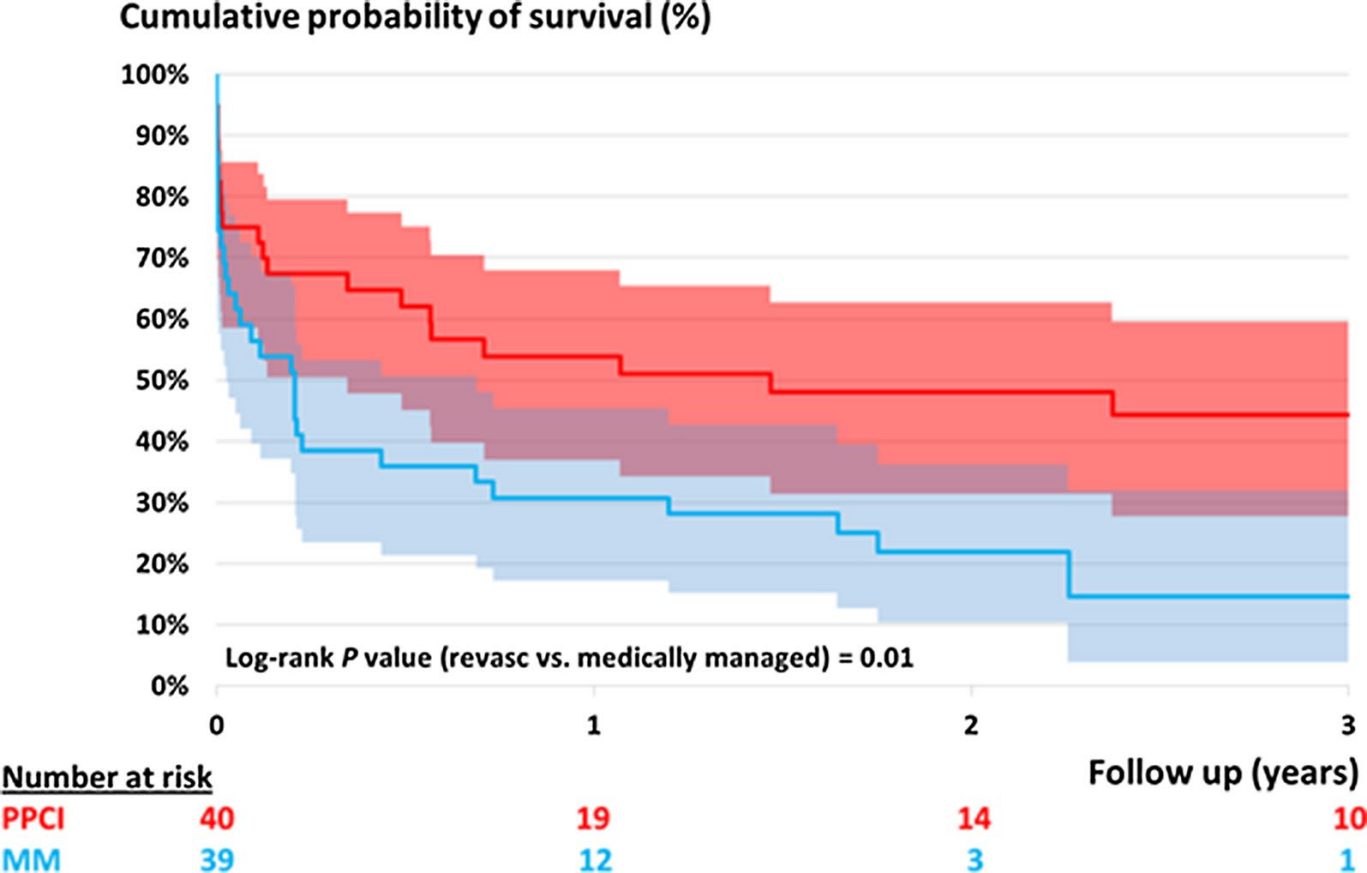
Kaplan Meier for all-cause mortality excluding completed infarcts

Figure 2 shows the Kaplan Meier chart for all-cause mortality, excluding completed infarcts.

The Kaplan Meier curves diverge immediately with a statistically significant difference in survival being reached by 3 years (44.3% vs 14.6%, *p* = 0.01).

Table 3 summarises the antiplatelet and anticoagulation therapy patients were treated with during admission.

**Table 3:** Antiplatelet/anticoagulation therapy

Variables	PPCI patients (n = 42)	MM patients (n = 69)	*P* value
*Anti-platelet agents*			
Aspirin only	0 (0)	9 (13.0)	**0.013**
Aspirin + Clopidogrel	10 (23.8)	32 (46.4)	**0.017**
Aspirin + Ticagrelor	31 (73.8)	15 (21.7)	** < 0.001**
Clopidogrel only	1 (2.4)	6 (8.7)	0.25
*Anticoagulation agent*			
Warfarin	1 (2.4)	2 (2.9)	> 0.99
Direct oral anti-coagulation	3 (7.1)	4 (5.8)	> 0.99
*Combination therapy*			
Clopidogrel + Anticoagulation	0 (0)	2 (2.9)	0.53
Triple therapy	4 (9.5)	4 (5.8)	0.47

Values in bold demonstrate statistically significant results (*p* < 0.05)

*PPCI* primary percutaneous coronary intervention, *MM* medically managed, Triple therapy aspirin + clopidogrel + anticoagulation

Values in brackets are (%)

The frequency of single antiplatelet usage was higher in the medically managed population. Ticagrelor was used in combination with aspirin more frequently in the PPCI group (73.8% vs 21.7% *p* < 0.001). Clopidogrel was used more frequently in the medically managed group. With those on anticoagulation, triple therapy was utilised more often than single antiplatelet plus anticoagulation, regardless of whether patients were treated with PPCI or medical management.

Table 4. summarises angiographic characteristics in the PPCI cohort in relation to 30-day mortality.

**Table 4:** Timing/target vessels in PPCI cohort and 30-day mortality

Variables	30-Day mortality (n = 10)	Discharged alive (n = 32)	*P* value
Symptom onset to balloon time (mins)	268.4 ± 234.2	242.9 ± 123.9	0.44
Door to balloon time (mins)	38.2 ± 13.3	37.2 ± 14.4	0.62
Cardiogenic shock	3 (30.0)	1 (3.1)	**0.036**
NSTEMI	1 (10.0)	0 (0)	0.24
*STEMI*	9 (90.0)	32 (100)	0.24
Anterior ECG changes	3/9 (33.3)	16 (50.0)	0.47
Inferior ECG changes	6/9 (66.7)	11 (34.4)	0.13
Lateral ECG changes	0 (0)	4 (12.5)	0.56
Posterior ECG changes	0 (0)	1 (3.1)	> 0.99
*Culprit vessel*			
LMS	0 (0)	1 (3.1)	> 0.99
RCA	6 (60.0)	11 (34.4)	0.27
LCx	0 (0)	4 (12.5)	0.56
LAD	4 (40.0)	15 (46.9)	> 0.99
Intermediate	0 (0)	1 (3.1)	> 0.99

Values in bold demonstrate statistically significant results (*p* < 0.05)

*PPCI* primary percutaneous coronary intervention, *NSTEMI* non ST elevation myocardial infarction, *STEMI* ST elevation myocardial infarction, *LMS* left main stem, *RCA* right coronary artery, *LCx* left circumflex artery, *LAD* left anterior descending artery

± values are mean ± standard deviation. Values in brackets are (%)

There were no differences in symptom-to-balloon or door-to-balloon times. Only one patient who presented as an NSTEMI was taken immediately for revascularisation. Cardiogenic shock was associated with a higher risk of 30-day mortality. No association was seen between infarct location by ECG changes or culprit vessel and mortality.

## Discussion

Nonagenarians represent an increasing cohort of patients as we have seen in our centre over the duration of this study. Post mortem studies have shown that coronary atherosclerosis is the leading cause of death in this population with as many as 33% dying as a result of acute myocardial infarction [9]. The decision to treat very elderly patients with STEMI medically is often made due to a combination of presumed lack of prognostic benefit and perceived risk from invasive management. A large Korean study demonstrated that PCI in nonagenarians was not associated with more bleeds or fatal in-hospital complications [10]. This was reflected in our cohort—there were no access related complications in any of the patients at our centre. Physician bias therefore may be leading to fewer nonagenarians receiving appropriate invasive therapy. The false activation rate was higher in the nonagenarian cohort as compared to overall at our centre between 2015 and 2019 (29.3% verses 17.6%). Although this does not reach statistical significance there is a clear difference between the groups which reflects the complexity of this study population.

In our population, a crude assessment of pre-admission functional status is reflected via NYHA classification, with no difference being seen between groups. Many patients were NYHA class I (64.3% in the PPCI group, 56.5% in the medically managed) reflecting a very healthy subset of nonagenarians. This is further demonstrated by the relatively low incidence of conditions such as dementia. The number of patients who lived in a supported living facility (nursing or residential home) can also be thought of as a surrogate for functional capacity. No statistically significant difference was evident between the medically managed and PPCI groups. Although not validated as a marker of frailty, the BCIS 30-day mortality score does demonstrate the pre-interventional risk [11]. The similarity in scores suggests patients managed medically were not significantly more co-morbid. However, those with an existing diagnosis of AF were managed conservatively more often. This may relate to poor rate control or a suspected embolic aetiology for myocardial infarction. The potential need for a period of triple therapy (with dual anti-platelets and anti-coagulation) and its associated bleeding risk may deter interventionalists considering PPCI. Direct oral anticoagulants and their relative safety may address this concern in the future [12, 13]. It is possible that a small minority of patients in the medically managed group had other non-ischaemic conditions associated with ST elevation. However, every patient included in this study had bedside echocardiograms confirming regional wall motional abnormalities and raised troponins consistent with localised infarct as opposed to Tako-Tsubo or pericarditis.

It is interesting to note that a quarter of patients in the medically managed group had atrial fibrillation but less than 9% were on anticoagulation. This may well suggest a perception of high bleeding risk and indeed increased frailty in this cohort. It should also be noted, that in this nonagenarian cohort, a large proportion of patients were treated with Clopidogrel (half of all medically managed and a quarter of those undergoing PCI). This is perhaps not surprising given that the regional protocol advocates the use of Ticagrelor as first line therapy unless there are cautions or contraindications. Again, this may reflect the perceived high bleeding risk.

We have demonstrated a statistically significant difference in mortality between those managed medically and those undergoing emergency coronary intervention. By 3 years, nonagenarians who had PPCI had similar survival to nonagenarians who had not had a myocardial infarction. The commonest reason to manage medically was late presentation. These patients were deemed to have completed infarcts and therefore unlikely to benefit from emergency coronary intervention. The large number of late presenters could be a confounding factor for our results. However, repeating the analysis with exclusion of completed infarcts demonstrated no change in the mortality benefit seen. Long term survival therefore does appear to improve when selected nonagenarians with STEMI are treated with coronary intervention.

Cardiogenic shock appears to predict poor prognosis in terms of 30-day mortality, as has been described previously and is to be expected [3]. Procedurally, no difference was detected between door-to-balloon time or symptom-to-balloon time indicating patients undergoing PCI all received similar quality care.

Appropriate selection of patients in this cohort is clearly important. However, there is little in the available literature that allows us to use an evidence-based approach to selecting the very elderly for PPCI. Accident and emergency departments almost certainly hold back the frailest patients and equally transfer the strongest, but there is a grey area in the middle who may be missing out on potentially life prolonging treatment. There is a need for validation studies using simple objective tools to allow clinicians to grade frailty systematically rather than relying on an “end of the bed” test. The Clinical Frailty Scale which was developed in Canada and validated in a large sample of elderly patients [14] is a mandatory assessment of frailty that is used in patients requiring left atrial appendage closure [15]. Its use in the acute coronary syndrome population has never been tested but would be the next logical step.

To our knowledge, this study represents the largest single cohort of nonagenarians activating the PPCI pathway. Our single centre study confers multiple benefits. The UK benefits from publicly available mortality data from the Office of National Statistics. Additionally, the single centre nature allows us to acquire a very comprehensive dataset in terms of morbidity and therapeutic intervention that is often not available in multicentre studies. Although selection bias is present, it alone could not account for the short- and long-term survival benefit seen. As people live longer, the incidence of nonagenarians presenting via the primary PCI pathway is sure to rise, our paper adds to the available literature confirming that age alone should not exclude anyone from treatment [16].

## Limitations

This was a retrospective, single centre study. Selection bias is inherent as peripheral hospitals are unlikely to refer the frailest nonagenarians and ambulance crews are unlikely to bring such patients to our centre directly. Moreover, the retrospective nature of the study led to opportunistic data collection, which increases the probability of unmeasured confounding factors (we did not for instance, have a complete dataset on geriatric assessments of frailty). The medically managed cohort were more co-morbid as evidenced by the higher incidence of atrial fibrillation and completed infarcts, as such differences observed may reflect increased mortality in the medically managed group rather than decreased mortality in the PCI group. We addressed this by repeating survival analyses excluding the completed infarct and found no difference in outcomes, however the size of our cohort limited our ability to perform any multivariate analysis and therefore the ability to draw firm conclusions. Prospective, randomised studies are unlikely to be conducted in this cohort of patients. However larger multi-centre studies where validated frailty scores are used, may help to better define the risk/benefit ratio. We did not collect information on bleeding complications outside of patient admission to our tertiary centre, however this has been previously covered in the existing literature [10].

## Conclusion

We present data that suggests in this series of select nonagenarians who presented with acute ST elevation myocardial infarction to our tertiary center, those undergoing primary percutaneous coronary intervention appeared to have a lower mortality compared to those managed medically.

## Supplementary information

**Additional file 1:** Supplemental File 1: NHS Research and Ethics Committee decision flowsheet.

## Data Availability

The datasets used and/or analysed during the current study are available from the corresponding author on reasonable request.

## Abbreviations

AF: atrial fibrillation
BCIS: British Cardiovascular Intervention Society
CK-MB: Creatine Kinase Myocardial Band
DGH: district general hospital
ECG: electrocardiogram
EF: ejection fraction
GFR: glomerular filtration rate
LAD: left anterior descending artery
LCx: left circumflex artery
LMS: left main stem
MM: medically managed
NSTEMI: Non ST elevation myocardial infarction
NYHA: New York Heart Association
ONS: Office for National Satistics
PCI: percutaneous coronary intervention
PPCI: primary percutaneous coronary intervention
RCA: right coronary artery
STEMI: ST elevation myocardial Infarction
